# A Survey to Identify the Current Management of Cow’s Milk Disorders and the Role of Goat Milk-Based Formulas in the Middle East and North Africa Region

**DOI:** 10.3390/nu14051067

**Published:** 2022-03-03

**Authors:** Wael A. Bahbah, Mostafa ElHodhod, Mohamed Salah, Fawaz AlRefaee, Muath AlTuraiki, Samira Mousa, Ali Al Mehaidib, Wafaa Helmi Ayesh, Ahmed N. El-Bazzar, Joseph El Haddad, Heba Y. El Khashab, Amr El Zawahry, Mohammed Hasosah, Sanaa Youssef Shaaban, Yvan Vandenplas

**Affiliations:** 1 Department of Pediatrics, Faculty of Medicine, Menoufia University, Shebin El-Kom 32511, Egypt; wael_bahbah@yahoo.com; 2 Faculty of Medicine, Ain Shams University, Cairo 11566, Egypt; mostafaelhodhod@yahoo.com; 3 Faculty of Medicine, October 6 University, Giza 12511, Egypt; 4 Egyptian Academy of Pediatrics, Cairo 11311, Egypt; mohamedsalahdr@yahoo.com; 5 Department of Pediatrics, Al Adan Hospital, Ministry of Health, Kuwait City P.O. Box 46969, Kuwait; fawazalrefaee@gmail.com; 6 Department of Pediatrics, King Salman Hospital, Riyadh 12769, Saudi Arabia; maturaiki@yahoo.com; 7 Medical Department, Faculty of Veterinary Medicine, Benha University, Benha 13518, Egypt; sameramousa@yahoo.com; 8 Department of Pediatrics, King Faisal Specialist Hospital & Research Center, Riyadh 11211, Saudi Arabia; mehaidib@kfshrc.edu.sa; 9 Department of Clinical Nutrition, Dubai Health Authority, Dubai P.O. Box 4545, United Arab Emirates; majdshahd@hotmail.com; 10 Department of Pediatrics, Ministry of Health Hospitals, Cairo 12613, Egypt; ahmed.n.elbazzar@gmail.com; 11 Department of Pediatrics and Neonatology, Saint George University Hospital, Beirut 1100, Lebanon; profhadad@gmail.com; 12 Department of Pediatrics, Sulaiman Al Habib Medical Group, Riyadh 12214, Saudi Arabia; heba241@hotmail.com; 13 Department of Pediatrics, Ain Shams University, Cairo 11566, Egypt; 14 Pediatrics Department, King’s College Hospital London, Dubai P.O. Box 340901, United Arab Emirates; picu2011@gmail.com; 15 Department of Pediatrics, Sharjah University, Sharjah P.O. Box 27272, United Arab Emirates; 16 King Abdullah International Medical Research Center, Pediatric Gastroenterology Department, National Guard Hospital, King Saud Bin Abdulaziz University for Health Sciences, Jeddah 21482, Saudi Arabia; myhasosah@yahoo.com; 17 Department of Pediatrics, Faculty of Medicine, Ain Shams University, Cairo 11566, Egypt; sanaanutrition@gmail.com; 18 KidZ Health Castle, UZ Brussel, Vrije Universiteit Brussel, 1090 Brussels, Belgium

**Keywords:** cow’s milk allergy, cow’s milk intolerance, goat milk-based formulas, Middle East and North Africa

## Abstract

Background: Cow’s milk allergy (CMA) and cow’s milk intolerance (CMI) are the major cow’s milk disorders observed in infants and young children. This study investigates, for the first time, physician knowledge regarding CMA and CMI prevalence, diagnosis, and management in the Middle East and North Africa (MENA) region. In addition, we explore the role of goat milk-based formula as an alternative in infants suffering from CMI. Method: This cross-sectional survey was conducted from December 2020 to February 2021. A convenience sample of 2500 MENA-based physicians received the questionnaire, developed by a working group of pediatric experts. Results: 1868 physicians completed the questionnaire, including pediatric specialists (80.8%), training physicians (0.2%), dermatologists (0.1%), family/general physicians (12.9%), neonatologists (3.6%), neurosurgeons (0.2%), allergy nurse specialists (0.3%), pharmacists (2.1%), and public health workers (0.1%). Differentiation between CMA and CMI was recognized by the majority of respondents (80.7%), for which the majority of respondents (35.4%) identified that the elimination and challenge test was the best test to differentiate CMA from CMI, whereas 30.7% and 5.4% preferred the immunoglobulin E (IgE) test and skin prick test, respectively. In addition, 28.5% of respondents reported that there is no confirmatory test to differentiate CMA from CMI. The majority of respondents (47.3%) reported that amino acid-based formula (AAF)/ extensively hydrolyzed formula (EHF) is the cornerstone for the management of CMA. However, most respondents (33.7%) reported that lactose avoidance was best for the management of CMI. Overall, 65% of the respondents were aware of nutritionally adapted goat’s milk formula as an alternative to cow’s milk products and 37% would recommend its routine use in infants (≤2 years of age). Conclusion: The results of this survey demonstrate that the majority of physicians are aware of the underlying pathophysiology and management of CMA and CMI. However, a significant proportion of physicians do not follow the clinical guidelines concerning CMA/CMI diagnosis and management. Notably, this survey identified that goat’s milk formulas may offer a suitable alternative to AAF/EHF in infants with CMI as they contain β-casein protein which is easily digestible. In addition, goat’s milk formulas contain higher levels of oligosaccharides and medium-chained fatty acids compared with standard cow’s milk formulas, yet further clinical trials are warranted to support the inclusion of goat’s milk formulas in clinical guidelines.

## 1. Introduction

Globally, cow’s milk is the most abundant mammalian milk consumed by humans, and cow’s milk-based formulas are commonly used to feed human infants [1,2]. As such, cow’s milk-based formulas are often the first food source introduced into non-breastfed infants. However, cow’s milk products are associated with the development of allergy, known as cow’s milk allergy (CMA), and gastrointestinal (GI) discomfort due to intolerance, known as cow’s milk intolerance (CMI) [3]. CMA and CMI are often clinically used interchangeably due to similar clinical presentation, yet CMA and CMI are discrete disorders which differ in etiology, prevalence, prognosis, and management strategies.

CMA is one of the most common food allergies in infants and young children, with a prevalence of 2–5% [4]. CMA is an immunologically mediated reaction to the cow’s milk proteins beta (ß)-lactoglobulin (the most abundant whey protein in cow’s milk) and casein which consists of several isoforms (i.e., αs1-casein, αs2-casein, k-casein, and β-casein) [4]. Clinical manifestations may be immunoglobulin E (IgE)-mediated with a rapid onset (i.e., within minutes to a couple of hours), or non–IgE-mediated with slow onset (i.e., within a couple of hours to days post ingestion). CMA specific clinical manifestations typically include, yet are not limited to, rashes, hives, swelling (lips and face), wheezing, tightness of the throat, and trouble swallowing. Other symptoms of CMA are also associated with CMI such as diarrhea, nausea, vomiting, abdominal cramps, bloating, gas, regurgitation, and reflux [5,6,7]. CMI differs from CMA as it arises from the body’s inability to digest specific components of cow’s milk such as proteins, lactose and fats [6,8]. The mechanism of action of CMI is yet to be elucidated, though one hypothesis is that CMI is caused by insufficient lactase enzyme activity [9]. However, recent studies, albeit in adult cohorts, demonstrated that bovine β-casomorphin-7 (BCM-7) released from β-casein A1 is an important contributor to CMI as it is associated with intestinal inflammation and exacerbated GI symptoms [9,10,11,12]. These symptoms are associated with A1 β-casein released BCM-7, an exorphin that activates μ-opioid receptors expressed throughout the gastrointestinal tract and body [10,11,13]. A recent study indicated increased GI symptoms associated with A1 β-casein compared with A2 β-casein [14]. In contrast, the β-casein A2 variant is hypothesized to release less BCM-7 and, thus, associated with less intestinal inflammation/GI symptoms compared with the A1 variant [9,10]. Interestingly, the β-casein in human and goat milk is categorized as “A2-like” due to similarities in β-casein structure and BCM-7 yield [10,11,12]. 

Dietary avoidance of cow’s milk is commonly recommended for non-breastfed CMA and CMI infants. Alternatives include modified versions of cow’s milk (extensively hydrolyzed formula [EHF]/partially hydrolyzed formula [PHF]), amino acid-based formula (AAF), plant-based formulas (e.g., soy protein and hydrolyzed rice-based formulas), and alternative mammalian milk options such as buffalo, sheep, and goat [15,16]. International guidelines recommend the use of EHF as an effective alternative to cow’s milk and AAF in infants intolerant to EHF or when presenting with severe symptoms [17,18,19,20]. However, in recent years there has been increased focus on the use of goat’s milk as an alternative to cow’s milk due to the presence of easily digestible “A2-like” β-casein. In addition, goat’s milk contains lower lactose content compared with cow’s milk (4.1 g versus 4.7 g per 100 g milk), and higher levels of both medium-chain fatty acids and oligosaccharides [21,22,23,24]. Importantly, several studies have demonstrated that the nutritional and growth profiles of infants fed with goat’s milk-based formulas are similar to those fed with cow’s milk-based formulas [25,26,27,28]. Furthermore, in vitro studies have shown that the kinetics of goat’s milk protein digestion closely resembled the profile of human milk protein digestion [1,28]. As such, there is a case for using goat’s milk-based formulas in CMA/CMI infants. 

Non-cow milk-based mammalian milks are nutritionally modified to mitigate the development of nutritional deficiencies [23,29,30]. Although the casein and whey percentages in goat’s milk are similar to cow’s milk (80% casein, 20% whey), efforts have been made to modify the blend to mirror whey-dominant human milk (40% casein, 60% whey) [31,32]. Furthermore, goat’s milk formulas often contain a modified fat blend utilizing a specific mixture of vegetable oils to achieve a high concentration of palmitic acid and mimic the composition/ function of palmitic acid present in breast milk [32,33]. Interestingly, research suggests that modification of fat in infant formulas may benefit the development of the gut microbiota by supporting the colonization of beneficial bacteria during early life [34,35]. 

To date, studies have been conducted to understand the epidemiology, diagnosis, and management of food allergies overall in Middle East and North African (MENA) infants [36,37,38,39,40,41]. However, no studies have been conducted to investigate physician knowledge on the prevalence, diagnosis, and management of CMA and CMI in the MENA region. The aim of this study is to evaluate physicians’ understanding of the prevalence, diagnosis, and management of CMA and CMI in the MENA region, and to identify the role of goat-milk based formulas in CMA/ CMI management. 

## 2. Materials and Methods

A questionnaire (see Appendix A) was developed by a working group of pediatrician experts. The working group comprised regional experts from various countries across the MENA region, including Prof. Yvan Vandenplas from Belgium. A face-to-face meeting was organized to reach a consensus on the questions and the final questionnaire was circulated electronically among 2500 healthcare professionals across the region. 

The survey was structured into two parts: the first focused on the respondents’ demographic and professional characteristics, and the second part included questions on CMA, CMI, and goat’s milk formulas. The survey aimed to assess physicians’ opinions on understanding the difference between CMA and CMI in terms of prevalence, diagnosis, and management, as well as using goat milk-based formula as an alternative to cow’s milk infant formula. Convenience sampling was employed to collect data. The survey was completed anonymously by pediatricians, general physicians, pediatric gastroenterologists, neonatologists, and other healthcare professionals predominantly in the Kingdom of Saudi Arabia, Egypt, Kuwait, United Arab Emirates, Lebanon, Iraq, and Yemen. Descriptive statistics were used to summarize the characteristics of the respondents and their responses to the questions.

## 3. Results

### 3.1. Physician Characteristics

1868 physicians completed the questionnaire (58.5% male, 41.5% female), including pediatricians (72.7% [*n* = 1358]), general physicians (i.e., an individual’s primary-care provider, 5.5% [*n* = 103]), family physicians (i.e., consultation provider to an entire family, 4.5% [*n* = 84]), neonatologists (3.6% [*n* = 67]), pediatric nutritionists (2.8% [*n* = 53]), and other healthcare workers (10.9% [*n* = 203]). The majority of physicians (41.9%, *n* = 782) were below 40 years of age and only 9.2% (*n* = 172) were over 60 years of age. A total of 46.1% (*n* = 862) participants worked in a government facility, 37.6% (*n* = 703) worked in a private facility, and 16.2% (*n* = 303) worked in both government and private facilities. Seventy-five percent (*n* = 1406) of the participants practiced in a hospital set-up and 23% (*n* = 424) had more than 20 years of experience in the pediatric arena. The characteristics of participating physicians are presented in Table 1.

### 3.2. Physician and Knowledge on Cow’s Milk Allergy and Cow’s Milk Intolerance

A total of 80.7% (*n* = 1508) of the respondents recognized that CMA and CMI were different disorders, while 19.3% (*n* = 360) believed that they were the same disorder. A majority (46.7%, *n* = 872) of the physicians reported the estimated prevalence of CMA (as per literature) to be 1–5%, whereas 24.5% (*n* = 457) of them estimated it to be 6–10%. As per clinical practice, 44.8% (*n* = 836) of the respondents reported the prevalence to be 1–5%. There were no notable differences in the responses of the participants regarding the prevalence of CMI as per literature and clinical practice (Table 2). Surprisingly, 23.7% (*n* = 442) and 30.2% (*n* = 565) of respondents were unaware of the prevalence data (as per literature) for CMA and CMI, respectively.

Most respondents (85.7%, *n* = 1601) reported that the allergic reactions to cow’s milk could be attributed to one or more of the cow’s milk proteins. Further, 47.5% (*n* = 887) reported that the most common age for non-allergic symptom onset associated with cow’s milk formula was 3 months and symptom resolution was 12 months. About 70% doctors (*n* = 1299) agreed that CMI may be related to the lipid, carbohydrate, or protein components of cow’s milk.

In clinical practice, 46.9% (*n* = 876) of respondents indicated that they perform specific-IgE tests in 1–25% of suspected CMA cases, whereas 31.7% (*n* = 593) of respondents reported that they do not conduct specific-IgE tests. When questioned about the best test to differentiate between CMA and CMI, 35.4% (*n* = 661) of the respondents favored the elimination and challenge test, 30.7% (*n* = 573) preferred the IgE test, and 5.4% (*n* = 101) chose the skin prick test. However, almost a quarter (28.5%, *n* = 533) of the respondents believed none of the tests were confirmatory to differentiate between the two cow’s milk-related disorders.

Regarding the cornerstone management approach for CMA patients, 47.3% (*n* = 883) of respondents reported that AAF/EHF are the foundation of standard practice, followed by EHF only (31.1% [*n* = 581]), nutritionally adapted goat’s milk formula (16.6% [*n* = 310]), and lactose avoidance (2.6% [*n* = 94]). On the other hand, 33.7% (*n* = 629) reported that lactose avoidance was the cornerstone management approach for CMI patients. Additional reported approaches included nutritionally adapted goat’s milk formula (26.3% [*n* = 491]), EHF only (23.1% [*n* = 431]), and AAF/EHF (17% [*n* = 317]). Interestingly, 65% (*n* = 1214) of the respondents were aware of nutritionally adapted goat’s milk formula and 26.3% (*n* = 491) considered it to be the cornerstone of CMI management in infants ≤2 years of age. However, only 37% (*n* = 691) of the respondents agreed that they would recommend it as routine treatment in infants ≤2 years of age. 

Protein digestibility was reported as the major difference between cow’s and goat’s milk formulas by 72.2% (*n* = 1349) respondents, and 35.8% (*n* = 669) of respondents recognized the role of goat’s milk-based formula for CMA management above fresh goat’s milk.

## 4. Discussion

This survey presents, for the first time, physicians’ knowledge on the prevalence, diagnosis, and management of CMA and CMI (beyond lactose intolerance) in the MENA region, and raises the beneficial applications of goat-milk based formulas in CMA/CMI management.

Based on the clinical practice of the survey respondents, our findings suggest that the estimated prevalence of CMA in the MENA region is between 1% and 5%. In comparison, European prospective studies have estimated the overall prevalence of CMA to be between 1.9% and 4.9% [42], which are consistent with the findings of this study [43]. Globally there is a paucity of comparable international epidemiological evidence regarding CMA and CMI prevalence data, which may be attributed to geographical differences in clinical evaluation. Available global prevalence rates of CMA and CMI were communicated with the respondents, yet a considerable proportion of physicians were unaware of the prevalence data (28% for CMA; 32.5% for CMI). 

The majority of respondents understood the pathophysiology of CMA and CMI and were aware of the gold standard approach to confirm an adverse reaction to cow’s milk protein, i.e., the oral challenge test [44]. The Middle East consensus guidelines published in 2014 consider the additional specific IgE measurement or skin prick test to confirm the diagnosis of CMA upon symptom assessment during physical examination, in parallel with an assessment of patients’ family history [45]. Notably, only half of the respondents reported the application of the specific IgE test for diagnostic confirmation in cases of suspected CMA. In comparison, 31% stated that there was no requirement for confirmatory IgE tests. These results suggest that a marked proportion of MENA-based physicians do not follow the guideline recommendations to diagnose CMA. Importantly, this highlights the need for initiatives to increase awareness and education regarding diagnostic approaches to improve patient prognosis. 

Survey respondents (47.3% [*n* = 883]) considered EHF/AAF to be the cornerstone of CMA management for non-exclusively breast fed infants. This is in line with the International and Middle Eastern consensus guidelines which recommend the use of EHF and AAF, alongside the elimination of cow’s milk formula and complementary foods containing cow’s milk protein [17,18,19,20,44]. However, the key considerations in the use of AAF/EHF in infants is the associated elevated cost and poor palatability compared with cow’s milk-based formulas. In addition, EHF is associated with reduced immunogenicity which may prevent the immune system from developing tolerance to milk proteins. Goat’s milk formulas are comparatively cheaper and better alternatives to EHF/AAF, providing similar benefits in the management of CMI in infants. However, it is important to note that goat’s milk formulas are typically 20–50% more expensive than standard cow milk-based formulas and there is a paucity of studies that directly compare cow’s with goat’s milk in CMA infants. Notably, the majority (65%) of respondents were aware of the availability of goat milk-based formulas, of which 26.3% considered it to the cornerstone of CMI management. However, only 37% of respondents agreed that they would recommend its use in infants below 2 years of age, which may be associated with the unavailability of goat milk-based formulas across MENA countries. These data also highlight the need for initiatives to promote goat’s milk formula awareness, utility, existing evidence, and overall build physician trust in the formula. Interestingly, many respondents considered nutritionally adapted goat’s milk-based formula as a treatment option for both CMA and CMI (CMA: 16.6% [*n* = 310], CMI: 26.3% [*n* = 491]). The overlap of treatment approaches employed for CMA and CMI, alike, suggests a lack of respondent understanding to differentiate between the two disorders. Though clinical differentiation between CMA and CMI presents a challenge for physicians since it is not always possible to distinguish them solely on clinical symptoms. Patch testing may be used to identify CMA from CMI, yet their application in MENA regions is not viable, possibly due to their limited application in hot climates. Further studies are needed to aid the identification and differentiation of CMA and CMI (beyond lactose intolerance) in dark skin populations based in hot climates. 

Nutritionally adapted goat’s milk formula is currently not recommended under clinical guidelines as an alternative to cow’s milk in the MENA region. However, goat’s milk protein is acknowledged by the European Food Safety Authority to be a suitable protein source for infants using follow-on formula. Interestingly, the majority of survey respondents (37% [*n* = 691]) reported that they would recommend routine use of nutritionally adapted goat’s milk formula in infants ≤2 years. This is accompanied by a growing body of evidence that has demonstrated nutritional and safety profile similarities between goat’s and cow’s milk formulas [25,26,27,28]. For example, a multicenter, randomized study demonstrated similar growth rates and nutritional status between infants fed with cow’s milk and goat’s milk formula [46].Studies have also demonstrated the favorable protein profile of goat’s milk/ goat’s milk-based formulas (i.e., A2 β-casein proteins, medium-chain fatty acids, and oligosaccharides). These components are associated with improved infant digestion compared with cow’s milk/ cow’s milk-based formulas [21,22,47]. In addition, studies have shown that the fat content in goat’s milk is an excellent source of energy for various metabolic processes [33]. Further, the high levels of oligosaccharides possess prebiotic properties, which are associated with the maintenance of a healthy gut microflora, immune development, and protection against gastrointestinal infections [24].

The findings of this study should be considered cognizant with the following limitations. This survey provides a perspective on the current opinion of physicians in selected countries from the MENA region, and their understanding of CMA and CMI in infants. However, the survey method using convenience sampling is limited by the non-random selection of participants. As such, the cohort of respondents may not represent all physicians in the respective country or region. In addition, the respondents self-selected responses, which may have introduced results bias. Further, the questionnaire was developed by a working group of pediatrician experts. A limitation of question 13 was identified after completion of the survey; the response selections did not include an option for whey proteins and limiting the casein intake. Future surveys may consider a revision of the response selections.

## 5. Conclusions

In conclusion, this study provides important insights regarding physicians understanding and perceptions of infant CMA and CMI in the MENA region. The increasing prevalence rates of CMA and CMI in the region, alongside the long-standing preconceived notion that CMI is restricted to lactose intolerance, emphasizes the need for physicians to follow local guideline recommendations to mitigate misdiagnosis. Since the use of guideline-recommended EHF/AAF is limited by high cost and reduced palatability, we propose and hypothesize that goat milk-based formulas offer a viable alternative to EHF/AAF formulas in infants with CMI. Goat’s milk-based formulas have been studied as alternatives to cow’s milk. Yet goat’s milk-based formulas remain underutilized, despite the favorable nutritional profile, protein profile, and growth outcomes among infants. The findings of this study indicate the need for physician initiatives to improve CMA/CMI awareness, understanding, diagnosis, and treatment knowledge to improve patient prognosis. In addition, there is a need to conduct further clinical trials to support the use of goat’s milk-based formulas in the management of CMI.

## Figures and Tables

**Table 1:** The demographic and self-reported characteristics of the survey respondents (*n* = 1868).

Characteristics	Demographic Data	Number	Percent
Gender	Male	1092	58.5%
	Female	776	41.5%
Age group	Less than 40 years	782	41.9%
	40–50 years	577	29.8%
	50–60 years	337	18%
	More than 60 years	172	9.2%
Specialty	Pediatrician (general)	1359	72.7%
	Pediatric gastroenterologist	47	2.5%
	Pediatric nutritionist	53	2.8%
	Pediatrician (other)	47	2.5%
	Family medicine	242	12.9%
	Physician (other)	8	0.4%
	Neonatologist	67	3.6%
	Pharmacist	39	2.1%
	Nurse specialist in allergy	6	0.3%
Country	Other (Algeria, Bahrain, India, Iraq, Jordan, Lebanon, Lybia, Oman, Pakistan, Sudan, Syria, Yemen)	90	4.8%
	Egypt	307	16.4%
	Kingdom of Saudi Arabia	10214	54.3%
	Kuwait	383	20.3%
	United Arab Emirates	67	3.6%
Place of practice	Private facility	703	37.6%
	Government facility	862	46.1%
	Both	303	16.2%
Years of experience in the pediatric arena	Less than 5 years	431	23.1%
	From 5 to 10 years	419	22.4%
	From 11 to 20 years	594	31.8%
	More than 20 years	424	23%
Facility of practice	Clinic	459	24.6%
	Hospital	1406	75.2%
	Maternal and child health centres	1	0%
	Primary healthcare centres	1	0%
	Store	1	0%

**Table 2:** Questionnaire results on physician knowledge of cow’s milk allergy (CMA) and cow’s milk intolerance (CMI).

Questions	Responses	*n* (%)
Q1: Are CMA and CMI the same disorder?	Yes	360 (19.3%)
	No	1508 (80.7%)
Q2: According to literature, the prevalence of CMA in an unselected population of infants is?	1–5%	872 (46.7%)
	6–10%	457 (24.5%)
	11–20%	97 (5.2%)
	I don’t know	442 (23.7%)
Q3: According to literature, the prevalence of CMI in an unselected population of infants is?	1–5%	316 (16.9%)
	6–10%	510 (27.3%)
	11–20%	477 (25.5%)
	I don’t know	565 (30.2%)
Q4: According to my personal experience in my practice, the prevalence of CMA in an unselected population of infants is?	1–5%	836 (44.8%)
	6–10%	527 (28.2%)
	11–20%	201 (10.6%)
	I don’t know	304 (16.3%)
Q5: According to my personal experience in my practice, the prevalence of CMI in an unselected population of infants is?	1–5%	579 (31%)
	6–10%	402 (21.5%)
	11–20%	598 (32%)
	I don’t know	289 (15.5%)
Q6: All the following are immune mediated allergy disorders in infants are below 2 years, except?	Cow’s milk allergy	109 (5.8%)
	Eosinophilic gastroenteritis	420 (22.5%)
	Lactose intolerance	1287 (68.9%)
	Atopic dermatitis	52 (2.8%)
Q7: In CMA, the allergic reaction is to one or more of the protein components present in cow’s milk?	True	1601 (85.7%)
	False	267 (14.3%)
Q8: CMI may be related as well to lipid, carbohydrate, or protein components of cow’s milk?	True	1299 (69.5%)
	False	569 (30.5%)
Q9: Which of the following describe the most common age of onset and resolution of non-allergic symptoms associated with cow’s milk-based infant formula?	3 months & 12 months	887 (47.5%)
	6 months & 36 months	318 (17%)
	9 months & 24 months	301 (16.1%)
	12 months & 48 months	362 (19.4%)
Q10: In a suspected case of CMA, how frequently do you request a “specific IgE test”?	Never	593 (31.7%)
	1–25% cases	876 (46.9%)
	26–50% of cases	208 (11.1%)
	More than 50% of cases	191 (10.2%)
Q11: The best test to differentiate between CMA and CMI is?	Elimination & challenge test	661 (35.4%)
	Skin prick test	101 (5.4%)
	IgE test	573 (30.7%)
	None is confirmatory (I don’t know)	533 (28.5%)
Q12: The cornerstone for management of CMA in formula fed infants (≤12 months old) is?	Lactose avoidance	94 (2.6%)
	Extensively hydrolyzed formula	581 (31.1%)
	Amino acid formula/extensively hydrolyzed formula	883 (47.3%)
	Nutritionally adapted goat’s milk formula	310 (16.6%)
Q13: The cornerstone for management of CMI in formula fed infants (≤12 months) is?	Lactose avoidance	629 (33.7%)
	Extensively hydrolyzed formula	431 (23.1%)
	Amino acid formula/extensively hydrolyzed formula	317 (17%)
	Nutritionally adapted goat’s milk formula	491 (26.3)
Q14: Are you aware of nutritionally adapted goat’s milk formula?	Yes	1214 (65%)
	No	654 (35%)
Q15: Would you recommend routine use of nutritionally adapted goat’s milk formula in infants ≤2 years?	Yes	691 (37%)
	No	630 (33.7%)
	I don’t know	431 (23.1%)
	Not available in my country	116 (6.2%)
Q16: The major difference in composition between cow’s milk and nutritionally adapted goat’s milk formula is?	Protein digestibility is better in nutritionally adapted goat’s milk formula compared to cow’s milk	1349 (72.2%)
	Oligosaccharides are present in higher concentration in cow’s milk than nutritionally adapted goat’s milk formula	110 (5.9%)
	Nutritionally adapted goat’s milk formula is cheaper than cow’s milk	57 (3.1%)
	Stool microbiota profile of cow’s milk formula fed infants is closer to breast fed infants than those fed with nutritionally adapted goat’s milk formula	352 (18.8%)
Q17: The difference between fresh goat’s milk & nutritionally adapted goat’s milk formula is?	Fresh goat’s milk intake results in folate deficiency	1006 (53.9%)
	Fresh milk intake is safe and recommended routinely for infants below 12 months of age	88 (4.6%)
	Goat milk-based formula has a role in treatment of cow’s milk allergy	669 (35.8%)
	Brucella and Q fever have been reported with the use of goat milk-based formula	105 (5.6%)

Legend: CMA: cow’s milk allergy; CMI: cow’s milk intollerance; IgE: Immunoglobulin E.

## Data Availability

Data can be requested from the Egyptian Academy of Pediatrics, ceo@egypediaacademy.com (Dr Mohamed Salah, CEO Egyptian Academy of Pediatrics).

## Appendix A. Questionnaire

Part 1—Demographics		
1. What is your specialty?	- General physician - Family physician - Pediatrician - Pediatric Gastroenterologist - Other (please specify)	
2. What country do you practice in?	a. Algeria	i. Kingdom of Saudi Arabia
	b. Bahrain	j. Kuwait
	c. Egypt	k. Oman
	d. Iran	l. Qatar
	e. Iraq	m. Tunisia
	f. Jordan	n. United Arab Emirates
	g. Lebanon	o. Other (please specify)
	h. Morocco	
3. What is your gender?	- Male - Female	
4. What is your age group?	- <40 years - 40–50 years - 50–60 years - >60 years	
5. Where do you practice?	- Government facility - Private facility - Both	
6. What facility do you practice in?	- Clinic - Hospital - Other (please specify)	
7. Do you work?	- Full time - Part time	
Part 2—Physician perception and knowledge of Cow’s Milk allergy (CMA) and Cow’s Milk Intolerance (CMI)		
1. Cow’s milk allergy & cow’s milk intolerance are the same disorder?	- Yes - No	
2. According to literature, the prevalence of cow’s milk allergy in an unselected population of infants is?	- 1%–5% - 6%–10% - 11%–20% - I don’t know	
3. According to literature, the prevalence of cow’s milk “intolerance” in an unselected population of infants is?	- 1%–5% - 6%–10% - 11%–20% - I don’t know	
4. According to my personal experience in my practice, the prevalence of cow’s milk allergy in an unselected population of infants is?	- 1%–5% - 6%–10% - 11%–20% - I don’t know	
5. According to my personal experience in my practice, the prevalence of cow’s milk “intolerance” in an unselected population of infants is?	- 1%–5% - 6%–10% - 11%–20% - I don’t know	
6. All the following are immune mediated allergy disorders in infants below 2 years, except?	- Cow’s milk allergy - Eosinophilic gastroenteritis - Lactose intolerance - Atopic dermatitis	
7. In cow’s milk allergy, the allergic reaction is to one or more of the protein components present in cow’s milk?	- True - False	
8. Cow’s milk intolerance may be related as well to lipid, carbohydrate, or protein components of cow’s milk?	- True - False	
9. Which of the following describe the most common age of onset and resolution of non-allergic symptoms associated with cow’s milk-based infant formula?	- 9 months & 24 months - 3 months & 12 months - 6 months & 36 months - 12 months & 48 months	
10. In a suspected case of CMPA, how frequently do you request a “specific IgE test"?	- Never - 1%–25% cases - 26%–50% of cases - More than 50% of cases	
11. The best test to differentiate between cow’s milk intolerance from cow’s milk allergy is?	- Elimination & challenge test - Skin prick test - IgE test - None is confirmatory (I don’t know)	
12. The cornerstone for management of cow’s milk allergy in formula fed infants (below 12 months) is?	- Lactose avoidance - Extensively hydrolyzed formula - Amino acid formula/extensively hydrolyzed formula - Nutritionally adapted goat’s milk formula	
13. The cornerstone for management of cow’s milk intolerance in formula fed infants (below 12 months) is?	- Lactose avoidance - Extensively hydrolyzed formula - Amino acid formula/extensively hydrolyzed formula - Nutritionally adapted goat’s milk formula	
14. Are you aware of nutritionally adapted goat’s milk formula?	- Yes - No	
15. Would you recommend routine use of nutritionally adapted goat’s milk formula in infants below 2 years?	- Yes - No - I don’t know - Not available in my country	
16. The major difference in composition between cow’s milk and nutritionally adapted goat’s milk formula is? (Choose one answer please)	- Protein digestibility is better in nutritionally adapted goat’s milk formula compared to cow’s milk - Oligosaccharides are present in higher concentration in cow’s milk than nutritionally adapted goat’s milk formula - Nutritionally adapted goat’s milk formula is cheaper than cow’s milk - Stool microbiota profile of cow’s milk formula fed infants is closer to breast fed infants than those fed with nutritionally adapted goat’s milk formula	
17. The difference between fresh goat’s milk & nutritionally adapted goat’s milk formula is (choose one answer please)?	- Fresh goat’s milk intake results in folate deficiency - Fresh milk intake is safe and recommended routinely for infants below 12 months of age - Goat milk-based formula has a role in treatment of cow’s milk allergy - Brucella and Q fever have been reported with the use of goat milk-based formula	
